# Successful ECMO-assisted open chest cardiopulmonary resuscitation in a postpartum patient with delayed amniotic fluid embolism

**DOI:** 10.1186/s40001-021-00628-1

**Published:** 2022-02-03

**Authors:** Yafen Wu, Jin Luo, Tao Chen, Hong Zhan, Jinfa Liu, Junxing Chen, Shouping Wang

**Affiliations:** Department of Anesthesiology, Third Affiliated Hospital, Guangzhou Medical University, No. 67, Duobao Road, Liwan District, Guangzhou, 510150 China

**Keywords:** Amniotic fluid embolism, ECMO, Open chest cardiopulmonary resuscitation, Puerperant

## Abstract

**Background:**

Amniotic fluid embolism (AFE) is a rare but potentially dangerous severe obstetrics complication, which is accompanied by an incidence between 1.9 and 6.1 per 100,000 births.

**Case presentation:**

Here, we report an AFE case after cesarean delivery diagnosed on a cardiac arrest complicated by acute respiratory distress syndrome and coagulopathy. Diagnosis, risk factors and pathophysiology for AFE have been fully discussed, besides, extracorporeal membrane oxygenation in the early management of cardiac arrest was used, describing the indication, efficacy and successful performed of open-chest cardiopulmonary resuscitation for the patient.

**Conclusion:**

In AFE with cute cardiovascular collapse, extracorporeal membrane oxygenation support can be considered as the alternative therapies.

## Highlights

1. The rare but dangerous amniotic fluid embolish after cesarean delivery was diagnosed.
2. A cardiac arrest accompanied by acute respiratory distress syndrome and coagulopathy was recorded.
3. Diagnosis, risk factors and pathophysiology for amniotic fluid embolish in this case were detailly discussed.

## Introduction

AFE is an obstetrical catastrophic syndrome, which occurs when amniotic fluid has entered maternal circulation causing respiratory failure, coagulation dysfunction and other pathological changes of obstetrical specific severe syndromes [1]. Despite rare, AFE remains one of the leading causes of direct maternal mortality (up to 86%) in high-income countries [2]. Therefore, collecting and reporting related cases is of great significance for guiding clinical practice. Here, we report an AFE case with cardiac arrest successfully treated by extracorporeal membrane oxygenation (ECMO) and open chest cardiopulmonary resuscitation (OCCPR). The entire surgical process has been restored to the greatest extent, in the hope of providing some basic references for clinicians. Besides, the patient has given permission to publish this case report.

## Case presentation

Due to placenta previa and suspected placenta accrete, a 35-year-old pregnant woman was admitted to the labor and delivery unit of this hospital at the 34 weeks and 5 days of gestation for a planned repeat cesarean section. The patient once underwent a cesarean section for premature rupture of fetal membranes, and the baby was delivered at the 41st week without complications. Afterwards, the patient was diagnosed of “autoimmune disease” because of positive anti-ro-52 anti-body. Dhydroxychloroquine, methylprednisolone and enoxaparin sodium were taken until the 34 weeks and 1 days of gestation, during which the patient's pregnancy was in vitro fertilization. At the 7th week of gestation, the patient had to be sent to a hospital prenatal care center because of a positive pregnancy test. Obstetrical ultrasonography with fetal survey performed after 24 weeks and 2 days of gestation revealed a normal fetal structure, sail-shaped placenta and low placenta situation. Besides, the patient was suggested not to have strenuous exercise and to seek immediate medical attention if vaginal bleeding were to occur. At the 28 weeks and 3 days of gestation, a normal 75 g oral glucose tolerance test result was confirmed. At the 34 weeks of gestation, obstetrical MR with uterus survey revealed a placenta that was positioned anteriorly with complete placenta previa, and possible placenta adhesion or placenta accrete were to occur. At the 34 weeks and 5 days of gestation, at about 03:50 am when the patient was sleeping, a massive blood flow through the vagina occurred without abdominal pain and vaginal fluid. At the same moment, the fetal movement was normal. Finally, the patient came to this hospital and was admitted to labor and delivery. From the examination in the labor and delivery unit: the pulse was 94 bpm (bpm), the oxygen saturation was 99%, the blood pressure was 130 mmHg over 90 mmHg and other vital signs were normal. Fetal heart rate became 145 bpm in a normal and regular rhythm. A speculum examination revealed a closed cervical and a small amount of bright red blood without pooling fluid and active bleeding.

Afterwards, the patient was admitted to the labor and delivery unit for observation. Notably, the patient felt well, but she is obese (weight: 87 kg, height: 156 cm) and is allergy to tinidazole. During daily life, she keeps away from alcohol, tobaccos and any illicit drugs. On the 3rd day after hospitalized, she continued to vagina bleeding, thus, plans were made immediately for a scheduled cesarean delivery considering the fetal and maternal safety. The blood type was B, Rh-positive with negative antibody testing. Hemoglobin level was 12.4 g/dL (reference range, 12.0–16.0), while the hematocrit was 33.9% (reference range, 36.0–46.0). Other laboratory test results were provided in Table 1.

**Table 1:** Laboratory data

Variable	Admission	Reference range
White-cell count (per mm^3^)	14,490	4000–10,000
Red-cell count (per mm^3^)	4,450,000	4,000,000–5,200,000
Hematocrit (%)	33.9	35–45
Hemoglobin (g/dl)	12.4	11.0–15.0
Platelet count (per mm^3^)	318,000	100,000–300,000
Prothrombin time (s)	10.5	9–14
Partial-thromboplastin time (s)	29.3	28–42
Prothrombin-time international normalized ratio	1.0	0.9–1.1
Fibrinogen (g/l)	3.69	2–4

That morning, the patient had to undergo cesarean section under general anesthesia due to placenta previa, suspected placenta adhesion or placenta accrete. The vital signs were normal under general endotracheal intubation anesthesia with rapid sequence intubation. A total of 175 mg of propofol with 50 mg of rocuronium and remifentanil 80 μg were rapidly infused. Meanwhile, with the help of direct laryngoscopy with clear view of tube passing the vocal cords, a size of 7.0 endotracheal tube was placed. Afterwards, the mechanical ventilation was initiated. Finally, 5% desflurane were inhaled and 0.1 μg/kg/min remifentanil were intravenously injected to guarantee anesthesia maintenance. Under ultrasound guidance, a central venous catheter and a radial artery catheter were inserted. To avoid uterine haemorrhage, bilateral ureteral catheterization under ureteroscopy were positioned. During the surgery, a vertical uterine incision was made through the placenta previa. At the 60 min after injection of the general anesthetic agent, a healthy boy was delivered, and the 1 min and 5 min Apgar scores were 8 and 9, respectively. Twenty units of oxytocin was drip administered immediately, then the placenta previa was removed completely. Luckily, operation went well leaving a structure-completed uterine. Thus, the obstetrician began to shut the abdomen, during which the bleeding was approximately 1500 ml and the hemoglobin level was 9.9 g/dL.

However, at the 84 min after delivery of the infant, as the abdominal fascia was being closed, the patient suddenly behaved ventricular tachycardia up to 196 bpm, and then turned to ventricular fibrillation. At the same time, the patient’s systolic blood pressure drastically fell to 32 mmHg over 22 mmHg, and the pulse became 0 bpm. Besides, oxygen saturation was as low as 60–70%. We immediately adopted cardiopulmonary resuscitation and intravenous lidocaine. Meanwhile, we compressed the chest continuously, and injected epinephrine to boost circulatory system together with using an icecap to protect the brain. About 10 min later, the blood pressure and electrical rhythm were restored, but this condition just maintains for ~ 5 min, and a repeated ventricular fibrillation occurred again surprisingly. Then, we implemented transthoracic echocardiography, while the patient was undergoing active resuscitation, and it revealed acute right ventricular failure, right ventricular dilatation and hypokines, and the EF is only 22%. Considering the marginal hemodynamic status and the severe cardiac dysfunction, together with 30 min cardiopulmonary resuscitation after the cardiac arrest, and large dose of inotropic agents, we chose to perform extracorporeal membrane oxygenation (ECMO) to support the cardiopulmonary circulation. ECMO is widely utilized in Covid patient with severe pulmonary dysfunction, it provides a new approach for clinical practice [1, 2]. Meanwhile, after continuous chest compressions and multiple electrical de-fibrillation, on many occasions, the patient can recovery to normal sinus arrhythmia, but few minutes later, ventricular fibrillation repeated occurred alternately. 144 min after delivery of the infant, cardiac arrest recurred again, the pulse decreased to 0 bpm, and oxygen saturation decreased to 60% accompanied by lowered blood pressure to 42/24 mmHg. However, even under continuous chest compressions and multiple electrical de-fibrillation, the heart rhythm was difficult to recover, and the cardiopulmonary circulation was even worse. At that time, patient ventricular fibrillation repeatedly, cardiopulmonary resuscitation had been carried out over 1 h after the first cardiac arrest, and myocardial damage from ischemia was increasing. Moreover, the maternal obesity, physiological anatomy changes in pregnancy with the diaphragm moved up and breast enlargement, resulting in worse and ineffective effect on cardiopulmonary resuscitation. For those reasons, an open chest cardiac massage had to be performed on the patient. After a 5 min administered intrathoracic cardiac compression, the sinus arrhythmia was restored. At the same time, the ECMO equipment started to run successfully, and most of the vasoactive agents could be discontinued, and the vital signs of the patient became stable gradually.

Unluckily, we found that the vaginal bleeding from the uterus were increasing, and the surgical incision began ozzing. The patient’s laboratory results (draw blood from 40 min after the first cardiac arrest).

demonstrated that thrombocytopenia, prolongation of prothrombin time (PT) and activated partial thromboplastin time (APTT), hypofibrinogenemia and d-dimer were all significantly extended or increased (Table 2). The patient was in a disseminated intravascular coagulopathy (DIC) situation. Considering the acute cardiopulmonary failure and disseminated intravascular coagulation which potentially resulted from AFE, and to control the massive haemorrhage and to improve coagulopathy, a hysterectomy was decided rapidly finally. Two hours later, the bleeding was finally controlled gradually.

**Table 2:** Values of PT, APTT, Hb and PLT throughout the process
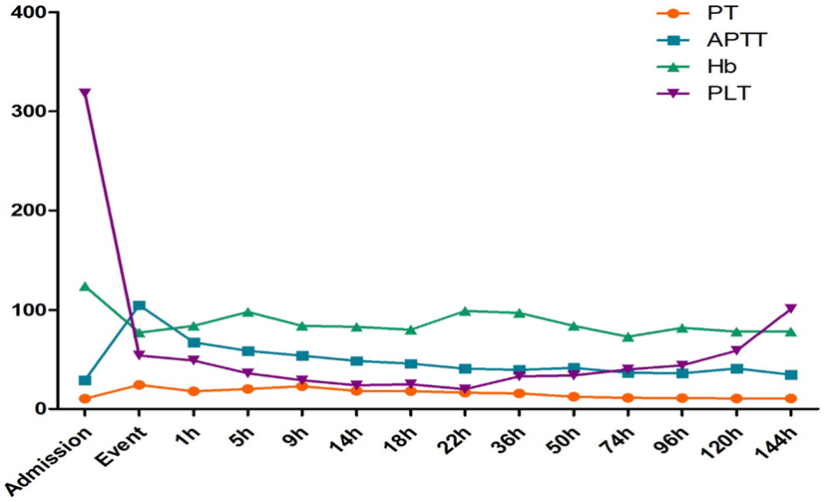

	Admission	Event	1 h	5 h	9 h	14 h	18 h	22 h	36 h	50 h	74 h	96 h	120 h	144 h
Fibrinogen, g/l	3.69	1.2	1.42	1.05	1.86	1.75	1.86	2.14	3.44	3.73	2.84	3.09	3.97	4.18
d-dimer, mg/l		33	27			12			9	10	7	3	3	6

After 4 h and 40 min of active resuscitation, the vital signs of the patient became stable gradually. By the end of this 8 h surgical procedure, the estimated blood loss was approximately 4800 ml. The blood products the patient received consisted of red-blood-cells-concentrated (24 units), fresh-frozen plasma (600 ml), normal frozen plasma (1600 ml), cryoprecipitate (10 units) and contractinogen (6 g).

**Fig. 1:**
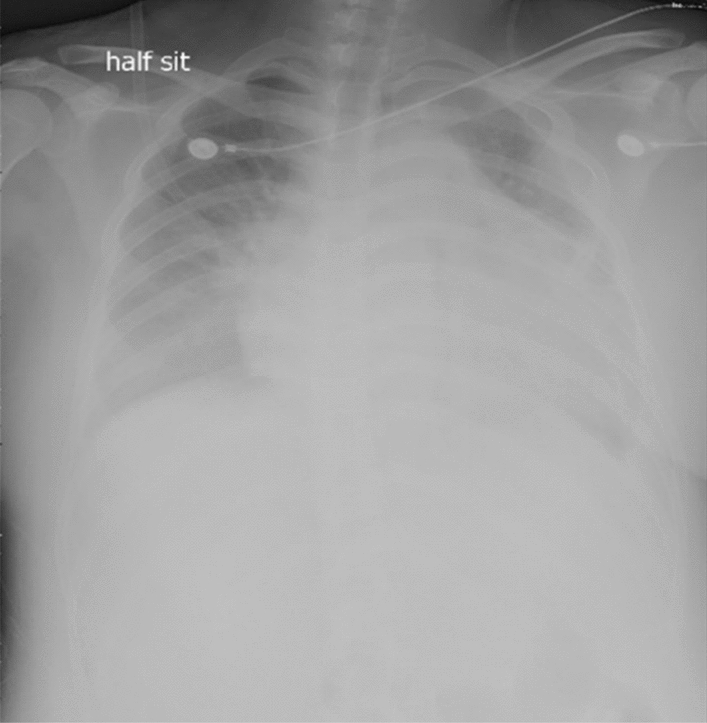
Postoperative chest X-ray from the ICU

The patient was then transported to the intensive care unit (ICU) for further monitoring with intubate and ECMO. Postoperative echocardiography did not indicate any evidence of pulmonary embolism (PE), but the left ventricular systolic function was decreased. Lower extremity Doppler ultrasound showed no deep venous thrombosis (DVT). The laboratory blood returns showed that PT and APTT were prolonged significantly, d-dimer was markedly elevated and platelets were decreased significantly. Chest X-ray was done to find patchy shadow in the left lung field (Fig. 1).

Gradually, the patient’s condition continued to improve under the advanced life support with ECMO and continuous renal replacement treatment (CRRT). 1 day after post-operation, the patient recovered her consciousness and could simply give a sign, but the circulatory function was still bad requiring continue to maintain the circulation with ECMO.

Under the support of ECMO, PT, APTT, and INR returned to normal levels gradually. The transthoracic echocardiography showed good recovery of right ventricular function. Fortunately, the ECMO was evacuated on postoperative day 4, and the tracheal catheter was pulled out on postoperative day 6. The patient was subsequently transferred out of the ICU to the medical center after the surgery 30 days, and she went back home without any cardiac complications and neurological sequelae after 90 days of hospitalization.

## Discussion

AFE is a rare but potentially dangerous severe obstetrics complication, and the incidence and mortality varied in various countries ~ 1.9–6.1 per 100,000 births. For this, perinatal mortality is reported from 61 to 86% in Europe and USA, respectively [3, 4]. AFE is one of the most feared diagnoses of obstetrical providers, which is also the second leading direct cause of maternal death in the USA and Europe [5, 6].

In general, AFE is an exclusion diagnosis without universal pathological or serological markers, it is based on clinical presentation for diagnosis, such as the classical triad of respiratory distress (apnea), cardiovascular collapse (hypotension) and coagulopathy [7]. In our report, this patient suddenly experienced cardiac arrhythmia during the operation, followed by cardiovascular collapse whose common causes of death in pregnant women such as heart disease, pulmonary thromboembolism, hemorrhage and anaesthesia are needed to be ruled out.

Peripartum cardiomyopathy can be considered as a cause of intraoperative cardiorespiratory collapse in pregnant women. The risk factors such as late stage of pregnancy, advanced maternal age and obesity can result in sudden cardiac arrests. However, the patient’s heart function was well and did not have symptoms, such as asthma or chest tightness throughout the pregnancy. Thus, peripartum cardiomyopathy should not be the reason. Besides, because the patient received general endotracheal intubation anesthesia, the explanation of high cephalad spread of a neuraxial anesthetic agent or local anesthetic poisoning induced sudden loss of consciousness and cardiac arrest can be ruled out. Meanwhile, hemorrhage and pulmonary embolism are the most common reasons for cardiac arrest during delivery in maternal. The patient had risk factors for hemorrhage, because she had a complete anterior placenta previa and suspected placenta accrete, but the placenta was delivered with ease, and the uterine tone was excellent, bleeding at the surgical site was well controlled with a little bleeding, so the hemorrhage can also be excluded. Here, can it be due to pulmonary thromboembolism? There are indeed risk factors, such as hypercoagulation during pregnancy, obesity and advanced maternal age. However, the intraoperative and postoperative ultrasound showed no deep-vein thrombosis, so no evidence of pulmonary thrombosis can be provided. Here, we have ruled out the possible diagnoses listed above, and based on the clinical presentation of this patient (sudden arrhythmia, hypoxemia, hypotension, hypocoagulation), AFE was finally diagnosed.

The pathophysiology of AFE remains incompletely understood. Previously, it was believed that the damage of physical barrier between mother and fetus can lead to AFE, including intracervix, injury of uterine damaged parts and placental attachment sites, uterine contraction pressure-assisted amniotic fluid into maternal circulation, resulting in mechanical obstruction of pulmonary vessels and further cardiac failure [8, 9]. However, now, AFE is more thought to be a maternal anaphylactic reaction called as “anaphylactoid syndrome of pregnancy” [3].

The featured characteristics of AFE includes a triad of sudden hypoxia and hypotension, followed in many cases by coagulopathy occurring in relation to labor and delivery [10]. In general, AFE can occur at any time during labor and delivery, as well as during the postpartum period. In most cases, it occurs at the 2 h before delivery or 30 min after delivery of placenta [7]. However, AFE may exist during the first or second trimesters of pregnancy, at the time of midtrimester induction of labor, amniocentesis, pregnancy termination or trauma [11]. The statistics data based on international reveal that 70% of cases of AFE occur during labor, 11% after a vaginal delivery, and 19% during a cesarean delivery after delivery of the infant [11].

AFE is often over-diagnosed in critically ill peripartum women, particularly when an element of coagulopathy is involved [12]. Different countries have different diagnostic criteria for the onset. International criteria for diagnosis of the USA and UK define the limit onset time to during labor or within 30 min of delivery of placenta [7], while in the Japan it is the symptoms appeared during pregnancy or within 12 h of delivery [12]. Here, this patient had sudden cardiovascular collapse moments after delivery of the placenta 84 min; therefore, this case was diagnosed as delayed amniotic fluid embolism.

Risk factors for AFE include increased maternal age, multiple gestations, cesarean delivery, placenta previa or placental abruption, cervical lacerations and uterine rupture [4]. In this case, she has older maternal age, placenta previa and suspected placenta accrete, and she has underwent a cesarean delivery. However, the AFE cannot be simply predicted or prevented with or without these risk factors. Therefore, for pregnant women with risk factors, the intraoperative monitoring and management should be strengthened and AFE should be considered in the differential diagnosis of a sudden cardiopulmonary collapse after excluding other likely causes.

Disseminated intravascular coagulation (DIC) is a common condition in AFE, it is reported to occur approximately 80% in patient with amniotic fluid embolism syndrome [13]. DIC may occur in conjunction with the cardiovascular collapse or in the later phases of the syndrome. Severe postpartum hemorrhage is closely related to DIC, and it is one of the main causes of maternal mortality. Therefore, a large-scale blood transfusion protocol, if available, should be started in the setting of uncontrolled hemorrhage, including platelets, fresh frozen plasma (FFP) and cryoprecipitate, fibrinogen is also important. This PT, APTT, and d-dimer values of the patient are increased abnormally approximately 40 min after cardiac arrest, we immediately initiated massive blood product and the patient gained a good outcome finally.

Early and high-quality cardiopulmonary resuscitation (CPR) is important and unique to save the life of both mother and the neonate in AFE. The quality of cerebral resuscitation is closely related to the patient’s residual permanent neurological damage. In this case, effective cardiopulmonary resuscitation was immediately implemented and given ice-cap brain for protection, but, due to the patient repeated sinus rhythm and ventricular fibrillation alternately, resulting the effect of cardiopulmonary resuscitation is worse and even ineffective, so an open chest cardiac massage (OCCM) was performed for the patient. Open-chest cardiopulmonary resuscitation (OCCPR) can trace back to 1870s, a distinguished physiologist called Moritz Schiff practiced OCCPR on drugs for the first time, and the surgeons called Paul Niehans was the first to attempt OCCPR on humans who had a cardiac arrest prior to undergoing general surgery [14]. According to statistic, traditional closed-chest cardiac massage (CCCM) only provides heart output in the range of 15–30% of precardiac arrest values, the myocardial and cerebral perfusion pressures were low [15]. Kornhall et al. demonstrated that standard OCCM can better maintain hemodynamic stability than CCCM, and the cardiac output during OCCM is twice that of traditional CCCM, which can significantly improve the survival rate and neurological outcomes of 72 h [16].

During the cardiopulmonary resuscitation of this patient, the ECMO was performed, because the patient continued to struggle with ventricular fibrillation and ongoing right ventricular failure. We chose venoarterial ECMO which provides both respiratory and circulatory support. We did not use heparin, because the activated clotting time (ACT) was more than 180 s. Under the support of ECMO, the patient’s cardiopulmonary system recovers quickly. The echocardiography ultrasound after 70 h of ECMO support showed the right ventricular systolic function was improved markedly. After 96 h of ECMO support, the ECMO was evacuated. The patient was discharged after 90 days.

## Conclusion

In conclusion, AFE is a rare but lethal obstetric complication. Early and aggressive management and treatment, high-quality cardiopulmonary resuscitation should be considered to save the life of both maternal and the fetal. Here, the surgical process have been carefully reviewed, whose aim is to provide some basic introductions to future critical maternal rescue.

## Data Availability

Not applicable.
